# Effect of motivational interviewing to promote advance care planning among palliative care patients in ambulatory care setting: a randomized controlled trial

**DOI:** 10.1186/s12904-025-01667-9

**Published:** 2025-01-31

**Authors:** Helen Yue-Lai Chan, Doris Yin-Ping Leung, Po-Tin Lam, Polly Po-Shan Ko, Raymond Wai-Man Lam, Kin-Sang Chan

**Affiliations:** 1 The Nethersole School of Nursing, Faculty of Medicine, The Chinese University of Hong Kong, 7/F Esther Lee Building, Shatin, New Territories, Hong Kong SAR, China; 2 School of Nursing, The Hong Kong Polytechnic University, Hong Kong SAR, China; 3 United Christian Hospital, Hospital Authority, Hong Kong SAR, China; 4 Kowloon East Cluster, Hospital Authority, Hong Kong SAR, China; 5 Haven of Hope Hospital, Hong Kong SAR, China

**Keywords:** Motivational interviewing, Advance care planning, Palliative care, Behavioral change, Medical decision

## Abstract

**Background:**

Many patients have mixed feelings about end-of-life care, even when facing life-limiting conditions. Motivational interviewing might be useful for supporting patients in evoking reasons for advance care planning. This study aimed to examine the effects of an advance care planning program adopting motivational interviewing among palliative care patients.

**Methods:**

A two-arm parallel randomized controlled trial was conducted between January 2018 and December 2019 in the palliative care clinics of two hospitals. Adult patients who were newly referred to palliative care services, with a score of 60 or higher in the Palliative Performance Scale and mentally competent, were eligible for the study. While all participants received palliative care as usual care, those in the intervention group also received the advance care planning program through three home visits. The primary outcome was the readiness to discuss and document end-of-life care decisions, and the secondary outcomes included decisional conflict, perceived stress, and quality of life.

**Results:**

A total of 204 participants (mean [SD] age, 74.9 [10.8]; 64.7% male; 80.4% cancer) were recruited. Generalized estimating equation analyses showed a significant improvement in readiness for advance care planning behaviors in the intervention group compared with the control group at 3 months post-allocation (group-by-time interaction, appointing proxy: *β* = 0.80; 95% CI, 0.25–1.35; *p* = .005; discussing with family: *β* = 0.76; 95% CI, 0.22–1.31; *p* = .006; discussing with medical doctors: *β* = 0.86; 95% CI, 0.30–1.42; *p* = .003; documenting: *β* = 0.89; 95% CI, 0.36–1.41; *p* < .001). The proportions of signing advance directives and placing a do-not-attempt cardiopulmonary resuscitation order were significantly higher in the intervention group, with a relative risk of 3.43 (95% CI, 1.55–7.60) and 1.16 (95% CI, 1.04–1.28), respectively. The intervention group reported greater improvements in social support and value of life than the control group immediately after the intervention. Significant improvements in decisional conflicts and perceived stress were noted in both groups.

**Conclusions:**

Motivational interviewing was effective in supporting patients to resolve ambivalence regarding end-of-life care, thereby increasing their readiness for discussing and documenting their care choices.

**Trial registration:**

ClinicalTrials.gov Identifier: NCT04162912 (Registered on 14/11//2019).

## Background

Advance care planning (ACP) empowers individuals to plan for future care through a series of actions, namely, clarifying individual’s end-of-life care preferences, relaying these preferences to significant others and health professionals, and documenting their care choices [1]. Although the importance of ACP is widely recognized, a considerable proportion of patients felt unready to consider future care [2–5]. Individual readiness for ACP is influenced by a variety of factors, including health literacy [6], emotions of patients and their family members [7, 8]. Hence, ambivalent feelings toward discussing end-of-life care matters are common [6, 8, 9].

Motivational interviewing is an evidence-based person-centered counseling approach designed to promote behavioral changes across different stages of change [10]. Motivational interviewing is widely utilized to motivate behavioral changes for health. Recently, motivational interviewing has also been integrated into the ACP process [11–13]. Both concepts underscore a person-centered empathetic orientation to facilitate reflection on personal values [14, 15]. The core concepts of motivational interviewing, which include partnership, acceptance, compassion, and evocation, align with the goal of ACP to empower individuals in planning for their future care [16]. In a feasibility study of ACP adopting motivational interviewing, patients with life-limiting conditions and their family members were willing to engage in discussions about end-of-life care, which they had previously found uncomfortable, because they felt understood in the process [17].

Although motivational interviewing promotes readiness for ACP, existing studies have primarily focused on certain behavioral outcomes, such as completion of advance directives or appointment of surrogates [11–13, 16]. The effects of motivational interviewing on other factors that explain the mechanism of the ACP process, such as decisional conflicts regarding end-of-life care and psychological outcomes, have yet been studied. Moreover, all these studies were conducted in the United States, leaving uncertainty regarding whether motivational interviewing is a culturally appropriate strategy for promoting ACP in Chinese communities, where discussing death-related matters is often considered offensive [5, 18]. Mixed feelings may arise during the medical decision-making process in the Chinese culture. Patients often follow the advice of physicians because they are viewed as authoritative figures given their professional training or make decisions based on family consensus. Ambivalence about end-of-life care may result if the decisions made do not align with their personal beliefs and values [19].

## Methods

### Study aim

This study aims to examine the effects of motivational interviewing for promoting ACP behaviors among palliative care patients. We hypothesized that the intervention group will exhibit significantly higher levels of readiness for ACP, lower levels of decisional conflicts regarding end-of-life care and stress, and better quality of life compared with the control group who received usual care.

### Design and setting of the study

This study was a prospective, two-arm randomized controlled trial conducted at two public hospitals in Hong Kong. The participants were recruited through palliative care outpatient clinics. The ethical approval for this study was obtained from the Kowloon East Cluster Research Ethics Committee and the New Territories East Cluster Research Ethics Committee. This study adhered to the reporting guidelines of the Consolidated Standards of Reporting Trials (CONSORT).

### Participants

Adult patients were eligible for the study if they had a diagnosis of a life-limiting condition and a Palliative Performance Scale (PPS) score ≥ 60% (i.e., reduced ambulation, activity, and self-care ability). PPS is a valid and reliable tool for assessing functional performance and predicting survival rates of palliative care patients. The median survival time of patients with a PPS of 60% ranged from 41 days to 65 days, regardless of diagnosis [20]. Patients were excluded if they were uncommunicable due to sensory or cognitive impairment, had language problems, were receiving active psychiatric treatment, or had advance directives or do-not-attempt cardiopulmonary resuscitation (DNACPR) order in the medical record.

The sample size was determined based on documentation of end-of-life care decisions, which was one of the ACP behaviors, as reported in our previous study of an ACP program among Chinese patients with advanced diseases [21]. The relative risk for participants communicating their end-of-life care preferences is approximately 3.0. A total of 204 patients was needed, with 102 per arm, to achieve 80% power and detect a 20% difference between the groups by using a two-sided chi-square test at a 5% significance level and an expected attrition rate of 30% in 3 months.

### Randomization and masking

A research assistant approached all newly referred patients to explain the purpose of the study and nature of the intervention and invite those who were interested to undergo eligibility screening. Consented and eligible patients were randomized in a 1:1 ratio to either the intervention group, which received motivational interviewing on planning for end-of-life care, or the control group, which received usual care, according to a randomization scheme. A statistician, who was not involved in the subject recruitment, generated a list of random numbers for group assignment and concealed the sequence using opaque sealed envelopes, which were opened by the research assistant during the group allocation process. The outcome assessor was blinded to the group assignments to avoid undue influence during the data collection process.

### Study arms

#### Intervention

The participants in the intervention group received a motivational interviewing-based ACP program on an individual basis. The intervention was adapted from an ACP grounded on narrative approach developed by the first author [21]. The three themes in the program, namely *My Stories*,* My Views and My Wishes*, were delivered through three one-hour weekly home visits within a month. Given that death-related matters are sensitive topics, building a trusting relationship between the ACP facilitator and the participants is a cornerstone for fostering in-depth discussion. Therefore, the program was designed to include repeated encounters for rapport building between the facilitator and the participants. In the first session, the participants were encouraged to share their illness experiences and beliefs about their health conditions and goals for future care. This approach allowed the facilitator to assess the individual understanding of the disease trajectory and readiness for ACP. In the subsequent two sessions, the facilitator provided tailored information to empower the participants to elicit intrinsic motivation for behavioral change. The multiple encounters highlight that ACP is a process of exploration and reflection of personal values. This enabled the participants to identify discrepancies between their preferred goal of end-of-life care and personal values, and any factors that supported or hindered their readiness to ACP. The participants’ views were recorded in a booklet for documentation. The participants could stop the discussion or withdraw from the study at any time.

The intervention protocol was reviewed by an expert panel, which included a palliative care specialist, a palliative care nurse, a social worker experienced in palliative care service, two nursing researchers who had used motivational interviewing in their research studies, a clinical psychologist experienced in delivering motivational interviewing, and a qualified trainer of motivational interviewing, to establish the content validity of the intervention. The intervention was delivered by a trained social worker experienced in using motivational interviewing. All intervention sessions were audio-recorded, with 20% randomly selected for an experienced motivational interviewing trainer to assess using the Motivational Interviewing Treatment Integrity (MITI) Coding Manual 4.2.1 revised in 2015 to ensure the treatment fidelity.

#### Usual care

All participants received usual care, which included a range of services offered by the palliative care teams, such as medical consultation at outpatient clinics, symptom control and psychosocial support provided by an outreach multidisciplinary team, and social or rehabilitation services offered at day care centers. All participants were provided an information leaflet explaining the concept and purpose of ACP and its potential benefits upon subject recruitment to standardize support for information needs about ACP during the study period across the two study groups.

### Measures

The study outcomes were measured at three time points: baseline (T0), 1 month (T1), and 3 months (T2) following group allocation. The primary outcome was readiness for four ACP behaviors at T2. Secondary outcomes included decisional conflict, perceived stress, and quality of life.

#### ACP behaviors

ACP behaviors in this study included four aspects: appointing a proxy for end-of-life care decisions, discussing end-of-life care with family, discussing end-of-life care with medical doctors, and documenting end-of-life care preferences. The participants were asked to rate their readiness for these behaviors using a five-point Likert scale, with scores ranging from 1 to 5 (where a high score indicates a high level of readiness). Furthermore, the participants’ medical records were reviewed at T2 to verify whether their end-of-life care decisions were documented using advance directives or a DNACPR order.

#### Decisional conflict

Decisional conflict regarding end-of-life care decisions was measured using the SURE test. There were four items assessing knowledge and support received for medical decision-making, with high scores indicating less decisional conflict [22].

#### Perceived stress

Perceived stress was measured by the 10-item Perceived Stress Scale (PSS). It comprises four positive items about coping and six negative items about affective reactions when people are confronted with uncontrolled or overloaded conditions, with high scores indicating a high level of stress [23].

#### Quality of life

Perceived quality of life was measured by the 23-item modified Quality of Life Concerns in the End-of-Life Questionnaire (mQOLC-E). It covers six domains, namely, physical, food-related, emotion, social support, value of life, and existential, rated on a four-point Likert scale, with high scores indicating enhanced quality of life [24].

### Data analysis

The study hypotheses were evaluated using intention-to-treat analyses, including all participants enrolled and randomized to each group. The baseline characteristics between completers and non-completers at 1 month and 3 months were compared using independent t-tests or chi-square tests. We used chi-square tests to assess the effect of the intervention and calculated the relative risk with a 95% confidence interval (CI) for the binary outcomes measured at T2. Generalized estimating equation (GEE) models were used to assess treatment effects from baseline to the follow-up assessments for continuous measures. The GEE models accounted for the dependence between repeated measures within individuals and can take into account of missing data. The interactions with each time variable of the treatment groups were assessed to examine whether the groups significantly differed with regard to changes in outcomes. Multiple imputation was used to impute missing values using chained equations in regression models that included outcome. A total of 500 imputed datasets were generated. The pooled parameter estimates from the GEE analyses and the corresponding 95% CI were calculated based on the 500 imputed datasets. The analyses were performed using SPSS version 28.0 (IBM, Armonk, NY, USA).

## Results

**Fig. 1:**
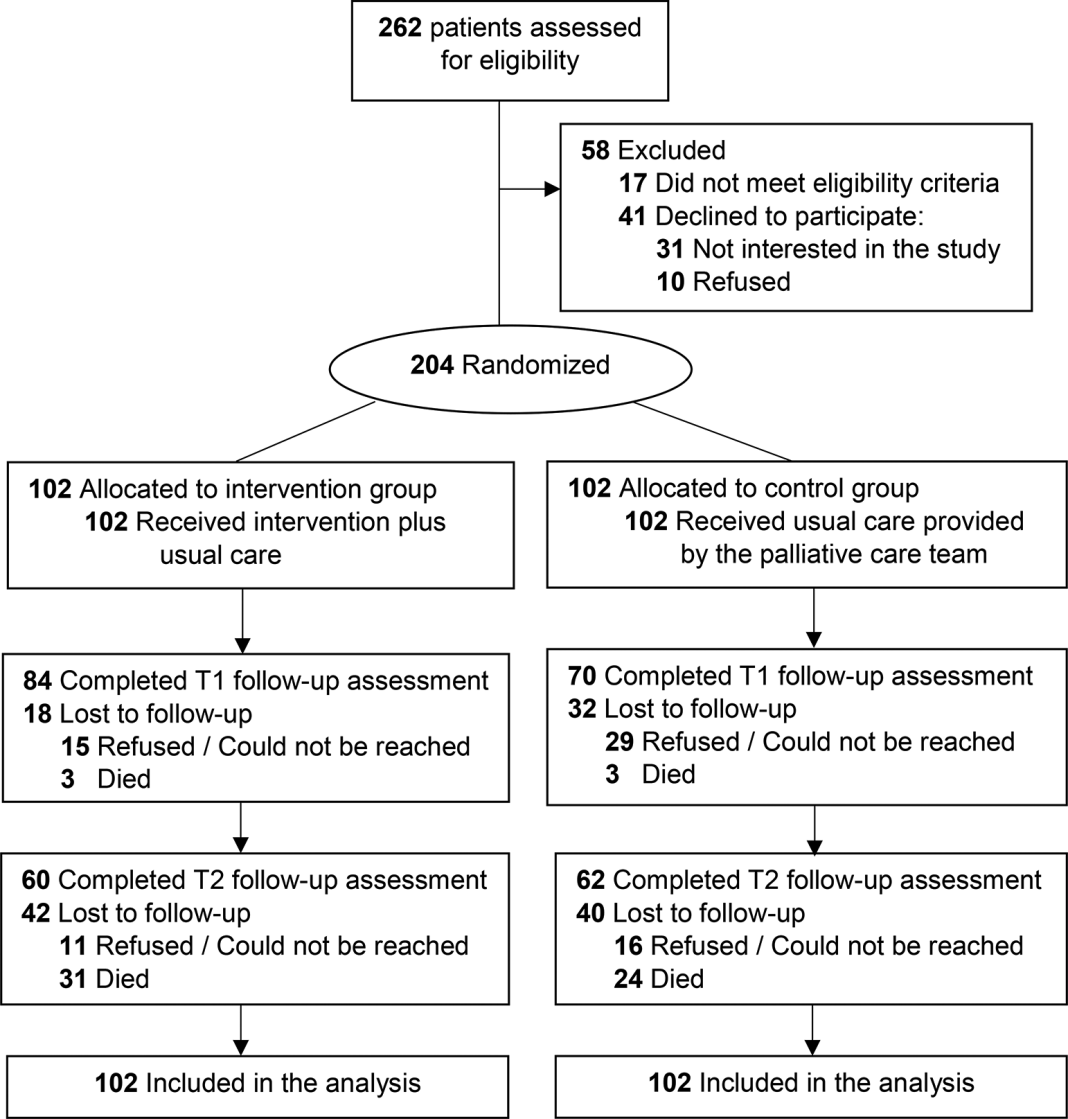
Participant flow diagram

Of the 245 patients who met the eligibility criteria, 204 (83.3%) consented to participate in the study (Fig. 1). The attrition rates at T2 were 41.2% for the intervention group and 39.2% for the control group. No significant difference in attrition rates was observed between the two groups.

Table 1 shows the demographic and clinical characteristics of the participants. The mean age was 74.9 (Standard Deviation [SD] 10.8; range, 40–96) years old. Majority of the participants were male (64.7%), married or cohabited with partners (65.7%), attained less than 6 years of education (71.1%), and living with their family (76.5%). The major diagnosis of the participants was cancer (80.4%). The mean score of the PPS was 66.6 (SD 12.1).

**Table 1:** Participants’ characteristics

Characteristics, *n*(%)	Total (*N* = 204)	Group	
		Intervention (*n* = 102)	Control (*n* = 102)
Age, mean (SD), years	74.9 (10.8)	75.8 (10.2)	73.9 (11.3)
Sex			
Male	132 (64.7)	66 (64.7)	66 (64.7)
Female	72 (35.3)	36 (35.3)	36 (35.3)
Educational level			
< 6 years	145 (71.1)	74 (72.5)	71 (69.6)
6–12 years	53 (26.0)	23 (22.5)	30 (29.4)
> 12 years	6 (2.9)	5 (4.9)	1 (1.0)
Marital status			
Single	6 (2.9)	1 (1.0)	5 (4.9)
Married / Cohabiting	134 (65.7)	73 (71.6)	61 (59.8)
Widowed / Separated	64 (31.4)	28 (27.5)	36 (35.3)
Living arrangement			
Alone	37 (18.1)	14 (13.7)	23 (22.5)
With family	156 (76.5)	83 (81.4)	73 (71.6)
In care homes	11 (5.4)	5 (4.9)	6 (5.9)
Diagnosis			
Cancer	164 (80.4)	81 (79.4)	83 (81.4)
Non cancer	40 (19.6)	21 (20.6)	19 (18.6)

Footnotes: All data are presented as number (percentage), unless specified

### Study outcomes

Tables 2 and 3 present the GEE results for the intervention effects on readiness for ACP behaviors and other secondary outcomes.

**Table 2:** Generalized estimating equation models for the intervention effects on the readiness for ACP behaviors

Readiness for ACP behaviors^a^	Mean (SD)		Time effect		Group*Time effect	
	Intervention (*n* = 102)	Control (*n* = 102)	β (95% CI)	*p*	β (95% CI)	*p*
Appointing proxy for EOL care decisions						
T0	2.00 (0.16)	2.24 (0.14)	/	/	/	/
T1	3.43 (0.17)	2.85 (0.18)	0.62 (0.22, 1.01)	0.002	0.81 (0.26, 1.36)	0.004
T2	3.84 (0.14)	3.28 (0.18)	1.04 (0.64, 1.44)	< 0.001	0.80 (0.25, 1.35)	0.005
Discuss EOL care with family						
T0	2.02 (0.16)	2.34 (0.14)	/	/	/	/
T1	3.46 (0.17)	3.04 (0.18)	0.70 (0.29, 1.11)	< 0.001	0.74 (0.19, 1.29)	0.008
T2	3.90 (0.14)	3.47 (0.18)	1.12 (0.72, 1.53)	< 0.001	0.76 (0.22, 1.31)	0.006
Discuss EOL care with medical doctors						
T0	2.03 (0.15)	2.22 (0.14)	/	/	/	/
T1	3.11 (0.17)	2.66 (0.18)	0.44 (0.03, 0.85)	0.034	0.64 (0.10, 1.18)	0.021
T2	3.84 (0.14)	3.17 (0.17)	0.95 (0.54, 1.37)	< 0.001	0.86 (0.30, 1.42)	0.003
Documenting EOL care preferences						
T0	1.70 (0.12)	1.81 (0.12)	/	/	/	/
T1	2.33 (0.16)	1.91 (0.15)	0.10 (-0.22, 0.42)	0.543	0.53 (0.09, 0.98)	0.019
T2	3.05 (0.17)	2.28 (0.17)	0.47 (0.10, 0.84)	0.013	0.89 (0.36, 1.41)	< 0.001

Footnotes: EOL, end-of-life; T0, baseline; T1, 1-month post-allocation; T2, 3-month post-allocation

**Table 3:** Generalized estimating equation models for the intervention effects on secondary outcomes

Outcomes	Mean (SD)		Time effect		Group*Time effect	
	Intervention (*n* = 102)	Control (*n* = 102)	β (95% CI)	*p*	β (95% CI)	*p*
SURE test						
T0	2.50 ± 0.18	2.58 ± 0.17	/	/	/	/
T1	3.67 ± 0.11	3.69 ± 0.11	1.11 (0.76, 1.45)	< 0.001	0.07 (-0.43, 0.56)	0.800
T2	3.63 ± 0.09	3.59 ± 0.10	1.01 (0.65, 1.37)	< 0.001	0.12 (-0.40, 0.64)	0.650
PSS						
*Positive items*						
T0	7.58 ± 0.35	6.46 ± 0.38	/	/	/	/
T1	5.59 ± 0.28	5.45 ± 0.27	-1.01 (-1.79, -0.23)	0.011	-0.97 (-1.99, 0.05)	0.061
T2	5.62 ± 0.28	5.21 ± 0.25	-1.25 (-2.04, -0.47)	0.002	-0.71 (-1.81, 0.40)	0.209
*Negative items*						
T0	5.78 ± 0.53	4.92 ± 0.51	/	/	/	/
T1	3.42 ± 0.35	3.75 ± 0.42	-1.18 (-2.11, -0.24)	0.013	-1.18 (-2.48, 0.13)	0.077
T2	3.37 ± 0.32	3.64 ± 0.40	-1.29 (-2.24, -0.33)	< 0.001	-1.13 (-2.53, 0.28)	0.117
mQOLC-E						
*Physical*						
T0	2.88 ± 0.08	2.96 ± 0.07	/	/	/	/
T1	2.81 ± 0.08	2.94 ± 0.08	-0.02 (-0.10, 0.13)	0.770	-0.05 (-0.25, 0.16)	0.658
T2	2.84 ± 0.09	2.91 ± 0.09	-0.05 (-0.24, 0.14)	0.611	0.01 (-0.26, 0.29)	0.921
*Food-related*						
T0	2.57 ± 0.08	2.74 ± 0.08	/	/	/	/
T1	2.56 ± 0.07	2.68 ± 0.08	-0.06 (-0.22, 0.10)	0.438	0.05 (-0.16, 0.27)	0.612
T2	2.73 ± 0.07	2.76 ± 0.09	0.02 (-0.1, 0.21)	0.863	0.15 (-0.13, 0.43)	0.298
*Emotional*						
T0	3.00 ± 0.09	3.26 ± 0.08	/	/	/	/
T1	3.21 ± 0.08	3.27 ± 0.09	0.01 (-0.13, 0.16)	0.861	0.20 (-0.01, 0.40)	0.059
T2	3.34 ± 0.08	3.35 ± 0.08	0.08 (-0.10, 0.27)	0.365	0.26 (-0.01, 0.53)	0.055
*Social support*						
T0	3.15 ± 0.06	3.24 ± 0.06	/	/	/	/
T1	3.33 ± 0.05	3.13 ± 0.06	-0.10 (-0.22, 0.01)	0.073	0.17 (0.05, 0.33)	0.043
T2	3.19 ± 0.06	3.19 ± 0.06	-0.05 (-0.20, 0.11)	0.556	0.08 (-0.14, 0.31)	0.461
*Value of life*						
T0	3.05 ± 0.07	3.23 ± 0.06	/	/	/	/
T1	3.25 ± 0.07	3.24 ± 0.07	0.03 (-0.12, 0.13)	0.963	0.20 (0.01, 0.39)	0.040
T2	3.23 ± 0.08	3.27 ± 0.07	0.04 (-0.13, 0.20)	0.678	0.16 (-0.10, 0.41)	0.233
*Existential*						
T0	2.96 ± 0.10	3.24 ± 0.08	/	/	/	/
T1	3.25 ± 0.09	3.29 ± 0.10	0.06 (-0.11, 0.23)	0.519	0.24 (-0.01, 0.47)	0.051
T2	3.35 ± 0.09	3.37 ± 0.09	0.14 (-0.07, 0.34)	0.193	0.26 (-0.05, 0.57)	0.097

Footnotes: PPS, Palliative Performance Scale; mQOLC-E, modified Quality of Life Concerns in the End-of-Life Questionnaire; T0, baseline; T1, 1-month post-allocation; T2, 3-month post-allocation

#### ACP behaviors

The readiness scores for all four behaviors increased in both groups, with significantly larger increments in the intervention group at T1 (appointing proxy: β = 0.81; 95% CI, 0.26 to 1.36; *p* = .004; discussing with family: β = 0.74; 95% CI, 0.19 to 1.29; *p* = .008; discussing with medical doctors: β = 0.64; 95% CI, 0.10 to 1.18; *p* = .021; documenting: β = 0.53; 95% CI, 0.09 to 0.98; *p* = .019). The intervention effects were sustained at T2 (appointing proxy: β = 0.80; 95% CI, 0.25 to 1.35; *p* = .005; discussing with family: β = 0.76; 95% CI, 0.22 to 1.31; *p* = .006; discussing with medical doctors: β = 0.86; 95% CI, 0.30 to 1.42; *p* = .003; documenting: β = 0.89; 95% CI, 0.36 to 1.41; *p* < .001). At T2, a significantly higher proportion of participants in the intervention group had signed an advance directive compared with the control group (23.5% vs. 6.9%), with a relative risk of 3.43 (95% CI, 1.55 to 7.60; *p* = .001). The proportion of participants with a DNACPR order recorded in the medical notes was also significantly higher in the intervention group than the control group (94.1% vs. 81.4%), with a relative risk of 1.16 (95% CI, 1.04 to 1.28; *p* = .006).

### Decisional conflict

The participants in both groups showed significant improvements in decisional conflict regarding end-of-life care at T1 and T2 (*p* values < 0.001) compared with T0. Nevertheless, the differences in the extent of improvement between the intervention and control groups were not statistically significant.

### Perceived stress

The participants in both groups also reported significant improvements in positive and negative dimensions of perceived stress at T1 and T2 compared with T0 (*p* values < 0.013). The group differences in the improvements also were not statistically significant.

### Quality of life

In terms of the quality of life, significantly greater improvements were observed in the social support domain (β = 0.17; 95% CI, 0.05 to 0.33; *p* = .043) and value of life domain (β = 0.20; 95% CI, 0.01 to 0.39; *p* = .040) for the intervention group at T1 compared with T0 than in the control group. However, the group differences were not sustained at T2.

## Discussion

This study is the first randomized controlled trial examining the adoption of motivational interviewing to facilitate ACP in Chinese palliative care patients. Our findings showed that motivational interviewing can significantly promote end-of-life care planning among palliative care patients in ambulatory care, compared with those who only received usual care. Although the control group reported comparable improvements in decisional conflict and perceived stress as the intervention group, these changes did not translate into ACP behaviors for the control group. This finding is consistent with Fried et al.’s study which found that fewer participants who only received written information about end-of-life care documented their care decisions compared to those who underwent motivational interviewing-based ACP [12]. This observation suggests that information giving can alleviate patients’ decisional conflicts on end-of-life care, but it is insufficient for encouraging them to complete ACP behaviors. The increase in decisional certainty without any behavioral change may explain the ambivalence experienced by patients in healthcare decision-making [3, 5, 9, 18]. This concurs with previous studies that advocate the use of motivational interviewing to increase the adoption of ACP behaviors [12, 16].

However, it is crucial to differentiate the purposes of motivational interviewing and ACP because these two concepts are fundamentally distinct [17]. Black and Helgason reminded that motivational interviewing is ethically appropriate in end-of-life care discussion only when a discrepancy between the client’s current behavior and his/her values or beliefs is identified [15]. The goal of motivational interviewing is not to pressure patients into completing ACP behaviors or to direct patients in refusing life-sustaining treatments. Rather, it equips ACP facilitators to identify mismatches between personal values and decisions made, while being attuned to emotional cues related to ambivalence about future care expressed by patients [8, 13]. The value of motivational interviewing in the ACP lies in helping individuals acknowledge their ambivalence towards end-of-life care, explore underlying reasons, and roll with resistance through adopting new behaviors. Studies have shown that patients are at varying stages of readiness for ACP behaviors, highlighting that ACP should be viewed as a process requiring multiple rounds of in-depth discussion [16]. Extended engagement provided time for patients to reflect on the inconsistencies between their beliefs and actions noted during the ACP process. The facilitators can then tailor their messages to support the uptake of ACP behaviors based on individuals’ desire, abilities, needs and reasons [25].

Our findings suggest that the value of our intervention is not limited to ACP behaviors. Notably, the ACP program did not induce stress or negative emotions in patients, but it improved their perceived social support and value of life, albeit briefly. This may be attributed to the presence and attentive listening of the facilitator during the ACP process. In addition to the roles of guiding patients to navigate different care options [16, 24, 26, 27], our findings reveal that ACP facilitator plays an important role in addressing patients’ psycho-existential needs. Literature has shown that individuals nearing the end of life often not being understood and struggle to share their views, leading to social withdrawal and loneliness [28, 29]. Therefore, the scope of ACP should extend beyond end-of-life care decision-making to include support for social connections and spirituality.

Consistent with other studies on motivational interviewing-based ACP [16], we found that a significantly higher proportion of participants in the intervention group completed advance directives at follow-up assessments compared to the control group. However, compared to other studies, the completion rate of advance directives was relatively low in our study [16]. The low completion rate may be due to structural barriers within the socio-legal context. Specific legislation recognizing the status of advance directives in Hong Kong was gazetted in 2024. This may explain the participants’ hesitance to sign an advance directive which was only recognized through common law framework at the time of the study [18]. Nevertheless, it appears that the participants communicated their care decisions with healthcare teams and had them documented in medical notes, including DNACPR orders. With the new legislation comes into effect, it is anticipated that more patients will be aware of their right to choices in end-of-life care. The integration of ACP into routine healthcare services has been widely supported [30, 31]. Our study serves as a timely example of how clinicians can build rapport with clients and identify ambivalence regarding end-of-life care through home visits or outpatient consultations in a structured format.

### Study limitations

We acknowledged several study limitations. First, the generalizability of the results may be limited to those who were more ready to plan for end-of-life care because participation to the study was voluntary. Despite this, the participation rate in our study was higher than that reported in an earlier study (23.7%) which adopted motivational interviewing for ACP delivered via telephone [12]. Another observation is the proportion of participants with non-cancer diagnosis was lower than those with cancer diagnosis. Second, the participants could not be blinded to the group allocation due to the nature of the intervention. Future research might consider providing attention control to the control group to equalize the interaction time for intervention.

## Conclusions

The findings of this RCT showed that motivational interviewing can significantly increase the readiness for discussing and documenting end-of-life care decisions among palliative care patients in ambulatory care settings, in addition to addressing their decisional conflicts and perceived stress. This intervention enables healthcare providers to support patients in identifying discrepancies between their values about end-of-life care and ACP behaviors in an empathetic manner.

## Data Availability

The datasets used and/or analysed during the current study are available from the corresponding author upon request.

## Abbreviations

ACP: Advance Care Planning
CI: Confidence Interval
DNACPR: Do Not Attempt Cardiopulmonary Resuscitation
GEE: Generalized estimating equation
MI: Motivational Interviewing
mQOLC-E: Modified Quality of Life Concerns in the End-of-Life Questionnaire
PPS: Palliative Performance Scale
