# Correction: Effect of motivational interviewing to promote advance care planning among palliative care patients in ambulatory care setting: a randomized controlled trial

**DOI:** 10.1186/s12904-025-01692-8

**Published:** 2025-03-14

**Authors:** Helen Yue-Lai Chan, Doris Yin-Ping Leung, Po-Tin Lam, Polly Po-Shan Ko, Raymond Wai-Man Lam, Kin-Sang Chan

**Affiliations:** 1 The Nethersole School of Nursing, Faculty of Medicine, The Chinese University of Hong Kong, 7/F Esther Lee Building, Shatin, New Territories, Hong Kong SAR, China; 2 School of Nursing, The Hong Kong Polytechnic University, Hong Kong SAR, China; 3 United Christian Hospital, Hospital Authority, Hong Kong SAR, China; 4 Kowloon East Cluster, Hospital Authority, Hong Kong SAR, China; 5 Haven of Hope Hospital, Hong Kong SAR, China

**Correction: BMC Palliat Care 24**,** 31 (2025).**

10.1186/s12904-025-01667-9

In this article [1] the author’s name Kin-Sang Chan was incorrectly written as Kin-Shan Chan.

The original article has been updated.
